# Stereotactic Body Radiation Therapy for Oligometastatic Breast Cancer: A Retrospective Multicenter Study

**DOI:** 10.3389/fonc.2021.736690

**Published:** 2021-10-28

**Authors:** Pauline Lemoine, Marie Bruand, Emmanuel Kammerer, Emilie Bogart, Pauline Comte, Philippe Royer, Juliette Thariat, David Pasquier

**Affiliations:** ^1^ Academic Department of Radiation Oncology, O. Lambret Center, Lille, France; ^2^ University of Lille, H. Warembourg School of Medicine, Lille, France; ^3^ Department of Radiation Therapy, Lorraine Institute of Oncology, Nancy, France; ^4^ Department of Radiation Oncology, Centre Francois Baclesse, Caen, France; ^5^ Biostatistics department, Oscar Lambret Center, Lille, France; ^6^ Department of Medical Physics, O. Lambret Center, Lille, France; ^7^ Advanced Resource Centre for Hadrontherapy (ARCHADE Research Community), Caen, France; ^8^ Laboratory of High-Energy Particle Physics, Institut National de Physique Nucléaire et de Physique des Particules, The National Engineering School of Caen (IN2P3/ENSICAEN), CNRS UMR 6534—Normandy University, Caen, France; ^9^ CRIStAL (Centre de Recherche en Informatique, Signal et Automatique de Lille [Research center in Computer Science, Signal and Automatic Control of Lille] UMR 9189, Lille University, Lille, France

**Keywords:** breast cancer, oligometastatic, stereotactic body radiotherapy (SBRT), Metastasis-directed therapy, progression free survival

## Abstract

**Introduction:**

Stereotactic radiotherapy may improve the prognosis of oligometastatic patients. In the literature, there is very little data available that is specific to breast cancer.

**Materials and Methods:**

We conducted a multicenter retrospective study. The primary objective was to estimate progression-free survival after stereotactic body radiotherapy (SBRT) using Cyberknife of breast cancer oligometastases. The secondary objectives were to estimate overall survival, local control, and toxicity. The inclusion criteria were oligometastatic breast cancer with a maximum of five lesions distributed in one to three different organs, diagnosed on PET/CT and/or MRI, excluding brain metastases and oligoprogressions. This was combined with systemic medical treatment.

**Findings:**

Forty-four patients were enrolled from 2007 to 2017, at three high-volume cancer centers. The patients mostly had one to two lesion(s) whose most widely represented site was bone (24 lesions or 44.4%), particularly in the spine, followed by liver (22 lesions or 40.7%), then pulmonary lesions (six lesions or 11.1%). The primary tumor expressed estrogen receptors in 33 patients (84.6%); the status was HER2+++ in 7 patients (17.9%). The median dose was 40 Gy (min-max: 15-54) prescribed at 80% isodose, the median number of sessions was three (min-max: 3-10). The median D50% was 42 Gy (min max 17-59). After a median follow-up of 3.4 years, progression-free survival (PFS) at one year, two years, and three years was 81% (95% CI: 66-90%), 58% (95% CI: 41-72%), and 45% (95% CI: 28-60%), respectively. The median PFS was 2.6 years (95% CI: 1.3 – 4.9). Overall survival at three years was 81% (95% CI: 63-90%). The local control rate at two and three years was 100%. Three patients (7.3%) experienced G2 acute toxicity, no grade ≥3 toxicity was reported.

**Conclusion:**

The PFS of oligometastatic breast cancer patients treated with SBRT appears long, with low toxicity. Local control is high. SBRT for oligometastases is rarely applied in breast cancer in light of the population in our study. Phase III studies are ongoing.

## Introduction

In women breast cancer ranks first in new cases of cancer and is the leading cause of cancer death (1). The concept of oligometastasis was described in 1995 as an intermediate stage between localized versus generalized disease, in which tumor extension is limited to a small number of metastases, generally less than five, commonly with one to two organ(s) affected (2). The *ESO-ESMO international consensus guidelines for advanced breast cancer (ABC 5)* (3) allows for, on the other hand, a maximum of five lesions to define oligometastatic disease, regardless of the number of organs affected. In breast cancer, this stage accounts for 1 to 3% of patients, even if the figures are not sufficiently representative (4).

Recently the ESTRO and EORTC have proposed a nomenclature for *de novo* recurrent or treatment-induced oligometastatic disease; this nomenclature must be validated in clinical trials or registries (5). Currently, the goal of local treatment for oligometastatic disease is to prevent the evolution of genetically unstable clones and to prevent further metastatic spread. The use of focal ablative therapies could potentially delay the introduction of systemic therapy, allow for a treatment pause in the case of fully controlled disease, or avoid an early change in treatment line.

The currently available focal therapies include surgery, which is the historical treatment for this condition, percutaneous thermal ablation, and radiation therapy. In the surgical series (6, 7), resections of secondary pulmonary or hepatic lesions were the most frequently performed surgeries in oligometastatic breast.

Regarding radiation therapy, occasional trials with generally small sample sizes have assessed the contribution of radiation therapy to the management of oligometastatic breast cancer. We can identify the prospective trial by Milano et al. (8), which enrolled 121 patients, including 39 cases of breast cancer. In 2018, Scorsetti et al. (9) enrolled 61 patients, including 11 cases of breast cancer. Among the published prospective studies, two trials conducted by Trovo et al. (10) and Milano et al. (11) in 2018 focused exclusively on breast cancer. They enrolled 54 and 48 patients, respectively. Two years progression free survival was 53% and 52% in these two trials respectively (8, 10).

The use of stereotactic radiotherapy will allow for the delivery of a high dose to the target for the purpose of ablation, while preserving more of the surrounding healthy tissue. Currently, the standard-of-care for oligometastatic disease in breast cancer is the use of systemic therapy, but the role of ablative therapies has not yet been clearly defined. The purpose of our study is to evaluate the contribution of stereotactic body radiotherapy to the management of breast cancer oligometastases in three high-volume Cancer Centers.

## Materials and Methods

### Trial Design and Patients

We conducted a multicenter retrospective cohort. Patients were enrolled from 2007 to 2017, at three cancer centers that participated in this study: the Lille Oscar Lambret Center, the Caen François Baclesse Center, and the Nancy Lorraine Institute of Oncology.

The inclusion criteria were patients over 18 years of age, managed for extra-cranial oligometastatic breast cancer with a maximum of five lesions distributed in one to three different organ(s), diagnosed by Computed Tomography (CT) in 24 patients (57,1%), Positon Emission Tomography - CT (PET-CT) in 28 patients (66,7%), and/or Magnetic Resonance Imaging (MRI) in 20 patients (47,6%). A bone scan was performed in 9 patients (21,4%). Histological confirmation was available in 21 patients (48.8%).

The exclusion criteria were patients with diffuse metastatic or oligoprogressive disease after chemotherapy, brain metastases, patients who received non-stereotactic radiation therapy, and patients treated with stereotactic radiation therapy after a metastasectomy or a local cementoplasty procedure.

### Treatment

The treatment was conducted using Cyberknife stereotactic radiotherapy from 2007 to 2017. Moving targets such as liver lesions were tracked by the *“*Synchrony*”* software, which allows the lesion to be tracked by placing fiducials near the tumour. For bone lesions, the patient was positioned using the *“*Xsight Spine*”* mode. This could be combined with systemic medical treatment (hormone therapy or chemotherapy more or less anti HER 2 therapy).

### Outcomes and Assessments

The primary endpoint was the progression-free survival (PFS)defined as the time interval from the start of SBRT to the date of the recurrence, or death from any cause. Patients alive without recurrence were censored at the date of last contact. The recurrences were identified by imaging. The secondary endpoints included overall survival (OS), local control and toxicity. OS was defined as the time interval from the start of SBRT until death from any cause. Patients alive were censored at the date of last contact. Local control was defined as the time interval from the start of SBRT to the date of the first local recurrence or other any recurrences, death from any cause were considered as a competitive event. The toxicities were graded using NCI-CTCAE scale in each centre by an experienced radiation oncologist. Severe toxicities were defined as ≥ grade 2 toxicities. Acute versus late toxicities were defined as toxicities occurring before or after 3 months after the end of treatment.

### Statistical Considerations

Conventional descriptive statistical methods (percentages, 95% confidence intervals, means, standard deviations, medians and ranges) were used to describe the patients characteristics and outcomes. The median follow-up and its interquartiles ranges was estimated by Schemper’s method (inversed Kaplan Meier). PFS and OS curves were estimated by the Kaplan Meier method. The survival rates with its associated 95% confidence intervals were estimated at 1 year, 2 years and 3 years. The percentage of patients who experienced toxicity was estimated overall as well as for acute and late toxicities. All statistical analyses were performed using Stata^®^ software, version 15.0 (StataCorp LLC College Station, USA).

## Results

Forty-four patients were enrolled. Their characteristics are presented in **Table 1**. Nineteen patients (52.8%) had systemic treatment, of which 13 received hormone therapy and 6 received chemotherapy. Data were missing for 8 patients.

**Table 1:** Demographics and baseline characteristics.

Characteristics (N = 44).	n	%	Characteristics (N = 44)	n	%
**Center**			**pT stage (MD=9)**		
Lille	22	50.0%	pT1a	4	11.4%
Nancy	15	34.1%	pT1c	8	22.9%
Caen	7	15.9%	pT2	16	45.7%
			pT3	7	20.0%
**Age at diagnosis (MD=1)**					
Median (range)	51	(31.0;79.0)	**pN stage (MD=8)**		
Average/standard deviation	53.4	12	pN0	13	36.1%
			pN1	16	44.4%
**Histological type (MD=1)**			pN2a	4	11.1%
NST	35	81.4%	pN3	3	8.3%
ILC	7	16.3%			
Other	1	2.3%	**HR Status (MD=5)**		
			ER+ PR+	23	59.0%
**cT stage (MD=13)**			ER+ PR-	10	25.6%
cT1a	1	3.2%	ER- PR-	6	15.4%
cT1c	5	16.1%			
cT2	12	38.7%	**HER2 status**		
cT3	12	38.7%	Negative	32	82.1%
cT4	1	3.2%	Positive	7	17.9%
					
**cN stage (MD=14)**			**Grade (MD=7)**		
cN0	15	50.0%	1	3	8.1%
cN1	12	40.0%	2	24	64.9%
cN3	2	6.7%	3	10	27.0%
cNx	1	3.3%			
			**Ki-67% (MD=21)**		
**cM stage (MD=7)**			Median - (range)	20	(3.0;90.0)
M0	16	43.2%	Average/standard deviation	22.8	18.3
M1	21	56.8%			
			**Vascular emboli (MD=18)**	11	42.30%
**Systemic treatment (MD=8)**	19	52,8%			
Chemotherapy	6	16,7%			
Hormonotherapy.	13	36,1%			

With MD, missing data; stage c, clinical stage; stage p, pathological stage; T, tumor; N, lymph node; M, metastasis; NST, no special type; ILC, invasive lobular carcinoma [sic]; HR, hormone receptors; ER, estrogen receptors; PR, progesterone receptors, and HER2, human epidermal growth factor receptor type 2.

The median follow-up of patients was 3.4 years with a 95% CI of 2.67-4.43 years.

The patients mostly had one to two lesion(s) whose most widely represented site was bone (24 lesions or 44.4%), particularly in the spine, followed by liver (22 lesions or 40.7%), then pulmonary lesions (6 lesions or 11.1%). The primary tumor expressed estrogen receptors in 33 patients (84.6%); the status was HER2+++ in 7 patients (17.9%). The median dose was 40 Gy (min-max: 15-54) prescribed at 80% isodose, the median number of sessions was three (min-max: 3-10). The median D50% was 42 Gy (min max: 17-59). The characteristics of the treatments are presented in **Table 2**.

**Table 2:** Characteristics of oligometastases treatments.

Characteristics (N = 44)	n	%
**SBRT treatment received**	**44**	**100.0%**
**Number of sessions (MD=1)**		
Median - (range)	3	(3.0;10.0)
Average/standard deviation	3.7	1.5
**Total dose (Gy) (MD=1)**		
Median - (range)	40	(15.0;54.0)
Average/standard deviation	36.6	10.4
**Prescription isodose (MD=1)**		
Median - (range)	80	(78.0;80.0)
Average/standard deviation	79.9	0.3
**PTV D2% (MD=17)**		
Median - (range)	49.3	(25.9;62.6)
Average/standard deviation	44.5	11.3
**PTV D50% (MD=4)**		
Median - (range)	42.4	(17.4;59.1)
Average/standard deviation	39.9	11.7
**PTV D98% (MD=21)**		
Median - (range)	36	(14.1;50.37)
Average/standard deviation	34.2	10.96

With Gy, Gray; PTV, planning target volume; Dx%, percent receiving dose ≥ x% of the volume (minimum dose covering x% of the concerning volume).

### Progression-Free Survival

At follow-up, 24 recurrences were identified, including 17 multimetastatic recurrences and seven oligometastatic recurrences. The latter did not occur at sites previously treated with radiation.

**Figure 1:**
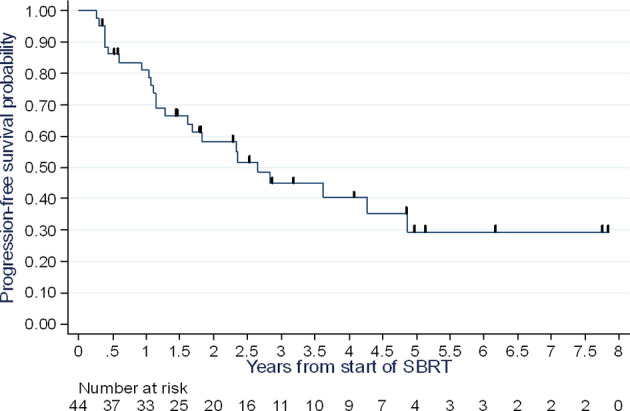
Progression-free survival assessment.

The PFS rate at one year was 81% (95% CI: 66-90%), at two years 58% (95% CI: 41-72%), and at three years 45% (95% CI: 28-60%), with a median of 2.65 years (range 1.28 – 4.87 years) (**Figure 1**).

### Overall Survival

At the end of follow-up, 10 of the 44 patients enrolled had died (22.7%); seven from their breast cancer (15.9%) and three from an unknown cause (6.8%).

**Figure 2:**
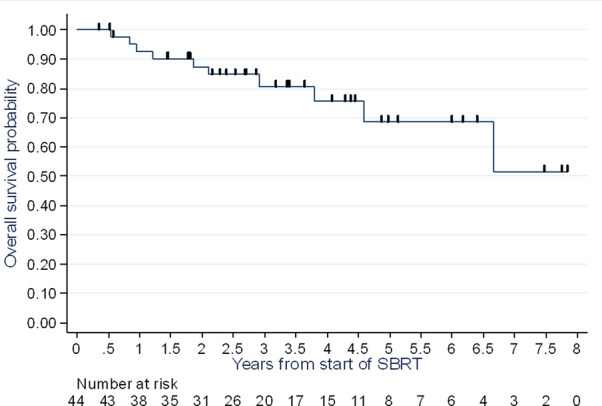
Overall survival assessment.

At one year, two years and three years, the overall survival rate was 93% (95% CI: 79-98%), 87% (95% CI: 72-95%), and 81% (95% CI: 63-90%), respectively (**Figure 2**).

### Local Control

Upon analysis of the data, we did not identify any recurrences at the sites treated with radiation, with a median follow-up of 3.4 years [95% CI 2.67-4.43 years].

### Toxicity Analysis

Ten patients (24%) experienced a maximum grade 1 acute toxicity and three patients (7%) experienced a grade 2 toxicity. No grade 3 or higher toxicities, either acute or late, were observed (**Table 3**).

**Table 3:** Maximum grade of toxicities per patient.

Toxicity (N=41, MD=3)	n	%
**Maximum grade (acute and late)**		
No toxicity	28	68.3%
Grade 1	10	24.4%
Grade 2	3	7.3%
**Maximum acute grade**		
No toxicity	28	68.3%
Grade 1	10	24.4%
Grade 2	3	7.3%
**Maximum late grade**		
No toxicity	40	97.6%
Unknown grade	1	2.4%

## Discussion

While the notion of oligometastasis is a relatively new concept and many authors have been interested in it, data specific to breast cancer is scarce in light of its incidence. The sample sizes remain low, and the prospective studies are few. To our knowledge, our study is among the few studies conducted exclusively on stereotactic radiotherapy for breast cancer oligometastases. This is one of the series with the largest population in this context. Indeed, most studies on the subject have heterogeneous populations, with inclusion of several patients with a primary or oligoprogressive disease. In our study, with a median follow-up of 3.4 years (95% CI 2.67-4.43), the PFS rate at two years was 58% (95% CI: 41-72%), and at three years 45% (95% CI: 28-60%), with a median of 2.65 years (range 1.28 – 4.87 years). Local control was 100%, with a median follow-up of 3.4 years [95% CI 2.67-4.43 years]. If we analyze our PFS data compared to prospective and retrospective published series, our results seem to align with them (**Tables 4** and **5**). In the trial by Trovo et al., PFS is evaluated at 75% at one year and 43% at two years with 54 patients enrolled (10). In the subgroup analysis of patients with breast cancer, Milano et al. reported, for 39 cases of breast cancer, a metastasis-free survival at 52% at two years and 36% at six years (11) The strength of our study therefore resides in its homogeneity, as well as the fact that the radiotherapy was exclusively performed in stereotactic conditions, the data from which was reported according to the recommendations in ICRU report 91 (18). However, our population did not allow us to perform subgroup analyses, in particular according to histological type. Indeed, the prognosis for metastatic disease differs based on the histology of the primary lesion. For example, patients with a triple-negative tumor have a worse PFS and overall survival than patients with luminal A or B carcinoma (19), and the potential role of stereotactic radiotherapy in these patients also remains to be determined. Scorsetti et al. report less promising results too due to the inclusion of only pulmonary and hepatic metastases, as well as patients with oligoprogressive disease (17).

**Table 4:** Review of the literature of retrospective series about SBRT for oligometastases of breast cancer.

Author	Primary	Definition	n patients	Follow-up	OS	PFS	LC
**Fumagalli et al.** (12)	Indifferent	≤5 sites	90	1 year	/	27%	84.5%
		Lung/Liver					
	(breast=8)			2 years	70%	10%	66.1%
**Mahadevan et al.** (13)	Indifferent	liver	427	Median	22 months	/	/
	(breast=42)			Breast	21 months	/	/
**Bhattacharya et al.** (14)	Indifferent	≤3 sites	76	1 year	84.4%	49.1%	/
	(breast=14)			2 years	63.2%	26.2%	/
**Onal et al.** (15)	Breast	≤5 sites	22	1 year	85%	38%	100% 88%
		Liver		2 years	57%	8%	
**Weykamp et al.** (16)	Breast	≤3 sites	46	2 years	62%	17%	89%
Our series	Breast	≤5 sites	44	1 year	93%	81%	100%
				2 years	87%	59%	100%
				3 years	81%	45%	100%

With T, follow-up time; OS, overall survival; PFS, progression-free survival; LC, local control; BM, bone metastasis.With T, follow-up time; OS, overall survival; PFS, progression-free survival; LC, local control; BM, bone metastasis.With T, follow-up time; OS, overall survival; PFS, progression-free survival; LC, local control; BM, bone metastasis.

**Table 5:** Review of the literature of different prospective trials on radiotherapy for oligometastases of breast cancer.

Author	Primary	Design	Definition	n patients	Follow-up	OS	PFS	LC
**Milano et al.** (8)	Indifferent	Prospective	≤5 sites	121	2 years	50%	/	/
	Breast	Single arm		39	2 years	74%	52%	87%
					6 years	47%	36%	87%
**Milano et al.** (11)	Breast	Prospective	≤5 sites	48				
		Single arm	BM	12	5 years	83%	/	/
					10 years	75%	/	/
			Non-BM		5 years	31%	/	/
					10 years	17%	/	/
**Scorsetti et al.** (17)	Breast	Prospective	≤ 3 sites	33	1 year	93%	48%	98%
			Liver/lung		2 years	66%	27%	90%
					3 years	/	/	90%
**Scorsetti et al.** (9)	Indifferent	Prospective	≤3 sites	61	3 years	33%	/	86.8%
	Breast	Phase II	Liver	11	5 years	20%	/	86.8%
		Single arm						
**Trovo et al.** (10)	Breast	Prospective	≤5 sites	54	1 year	/	75%	/
		Phase II	SBRT or IMRT		2 years	95%	53%	97%

With OS, overall survival; PFS, progression-free survival; LC, local control; SBRT, stereotactic body radiotherapy; IMRT, intensity modulated radiotherapy; BM, bone metastasis.

The phase 2 randomized trial SABR COMET enrolled 99 varied oligometastatic patients with primary tumors regardless of treatment with stereotactic body radiotherapy; 18 patients had an breast oligometastatic cancer. The mean overall survival was 28 months in the control group and 41 months in the SBRT group (20). Recently, in a prospective registry that included 1,472 patients treated with SBRT for oligometastatic disease, only 78 patients had breast cancer. The local control and metastasis-free survival at two years was respectively 82% (95% CI: 69-90%) and 52% (95% CI: 47-56%) (21). In our series local control was 100%, probably related to very hypofractionated regimen consistent with low alpha/beta ratio of breast cancer. In SABR COMET the regimen was 30-60 Gy in 3-8 fractions and local progression was a component of failure in 21% of failures in the SBRT arm (20).

Our population, in three high-volume Cancer Centers (a total of approximately 2,900 new patients treated annually for localized breast cancer), may seem small and can be explained in several ways: exclusion of brain metastasis as well as patients with oligoprogressive disease, but also by the fact that oligometastatic patients are rarely referred to radiotherapy and almost exclusively receive a first-line chemotherapy or hormone therapy. Finally, one of the limitations of our study is the retrospective nature, which gives it limited statistical power

These data seem supportive of SBRT in these patients nevertheless the benefit will be specified by ongoing randomized trials. In all these series, including ours, systemic therapy was associated with SBRT, which probably influenced PFS. It’s important to note that all ongoing trial evaluating SBRT in these patients compare systemic treatment with or without SBRT. Currently it seems too early to evaluate SBRT without systemic treatment in patients who can benefit from it.

Currently, several phase III trials are open (22), including the trials SABR-COMET (23), STEREO-OS (24), STEREOSEIN (25) and NRG BR002 (26). However, apart from STEREOSEIN and NRG BR002, these prospective “pantumor” trials may not be able to make a conclusion about the value of this strategy based on primary tumor site and tumor phenotype. Trials including a sufficient number of breast cancer patients, classified by histology, will help clarify the potential benefit by molecular subtypes.

SBRT may have a pro-immunogenic effect. The immune response and the combination of this treatment with immunotherapy and the immune response deserve further investigation (27).

## Conclusion

The current management of oligometastatic breast cancer relies primarily on medical management with systemic therapy. Local treatments such as radiation therapy are used for symptomatic purposes. SBRT for oligometastases is rarely applied in breast cancer in light of the population in our study. In our study, the PFS of oligometastatic breast cancer patients treated with stereotactic body radiotherapy appears long, with low toxicity, whereas systemic treatment may have contributed to PFS. Local control is high. The few published studies seem to show a benefit in treatment of breast cancer oligometastases with stereotactic radiation, however prospective studies dedicated to this type of cancer are needed to clarify the potential benefit according to molecular subtypes.

## Data Availability Statement

The raw data supporting the conclusions of this article will be made available by the authors, without undue reservation.

## Ethics Statement

Ethical review and approval was not required for the study on human participants in accordance with the local legislation and institutional requirements. Written informed consent for participation was not required for this study in accordance with the national legislation and the institutional requirements.

## Author Contributions

Study conception and design: DP and PL. Data collection: PL, MB, EK, EB, PC, PR, JT, and DP. Data analysis and interpretation: PL, MB, EK, EB, PC, PR, JT, and DP. Statistical analysis: EB. All authors contributed to the article and approved the submitted version.

## Conflict of Interest

The authors declare that the research was conducted in the absence of any commercial or financial relationships that could be construed as a potential conflict of interest.

## Publisher’s Note

All claims expressed in this article are solely those of the authors and do not necessarily represent those of their affiliated organizations, or those of the publisher, the editors and the reviewers. Any product that may be evaluated in this article, or claim that may be made by its manufacturer, is not guaranteed or endorsed by the publisher.
